# HIV-1 Transcription Inhibition Using Small RNA-Binding Molecules

**DOI:** 10.3390/ph17010033

**Published:** 2023-12-25

**Authors:** Pooja Khatkar, Gifty Mensah, Shangbo Ning, Maria Cowen, Yuriy Kim, Anastasia Williams, Fardokht A. Abulwerdi, Yunjie Zhao, Chen Zeng, Stuart F. J. Le Grice, Fatah Kashanchi

**Affiliations:** 1 Laboratory of Molecular Virology, School of Systems Biology, George Mason University, Manassas, VA 20110, USA; pkhatkar@gmu.edu (P.K.);; 2 Institute of Biophysics and Department of Physics, Central China Normal University, Wuhan 430079, China; 3 Basic Research Laboratory, National Cancer Institute, Frederick, MD 21702, USA; 4 Physics Department, The George Washington University, Washington, DC 20052, USA

**Keywords:** HIV-1 transcription, transactivation response element (TAR) RNA, transcription elongation factor-b (P-TEFb), SWI/SNF complex, molecular modeling, molecular simulation

## Abstract

The HIV-1 transactivator protein Tat interacts with the transactivation response element (TAR) at the three-nucleotide UCU bulge to facilitate the recruitment of transcription elongation factor-b (P-TEFb) and induce the transcription of the integrated proviral genome. Therefore, the Tat–TAR interaction, unique to the virus, is a promising target for developing antiviral therapeutics. Currently, there are no FDA-approved drugs against HIV-1 transcription, suggesting the need to develop novel inhibitors that specifically target HIV-1 transcription. We have identified potential candidates that effectively inhibit viral transcription in myeloid and T cells without apparent toxicity. Among these candidates, two molecules showed inhibition of viral protein expression. A molecular docking and simulation approach was used to determine the binding dynamics of these small molecules on TAR RNA in the presence of the P-TEFb complex, which was further validated by a biotinylated RNA pulldown assay. Furthermore, we examined the effect of these molecules on transcription factors, including the SWI/SNF complex (BAF or PBAF), which plays an important role in chromatin remodeling near the transcription start site and hence regulates virus transcription. The top candidates showed significant viral transcription inhibition in primary cells infected with HIV-1 (98.6). Collectively, our study identified potential transcription inhibitors that can potentially complement existing cART drugs to address the current therapeutic gap in current regimens. Additionally, shifting of the TAR RNA loop towards Cyclin T1 upon molecule binding during molecular simulation studies suggested that targeting the TAR loop and Tat-binding UCU bulge together should be an essential feature of TAR-binding molecules/inhibitors to achieve complete viral transcription inhibition.

## 1. Introduction

It is challenging to treat retroviruses, which integrate their genome into the host’s DNA and cause various pathologies, many of which are life-threatening. HIV-1 is the most transmitted retrovirus among humans. As of 2021, 38.4 million individuals were living with human immunodeficiency virus type 1 (HIV-1) with 1.5 million new infections per year [1]. HIV-1 is a retrovirus that weakens the immune system, enabling opportunistic infections and the onset of acquired immunodeficiency syndrome (AIDS). The current measure of care is combination antiretroviral therapy (cART) drug cocktails, which significantly reduce viral loads in circulation by blocking practically all phases of the virus cycle, from viral entry into the host cell to viral budding from the infected cell [2]. However, interruption of the cART regimen can lead to viral rebound due to poor protocol adherence or the generation of drug-resistant strains [3,4,5,6], as well as cognitive impairment due to HIV-associated neurocognitive disorders (HAND) in long-term controlled patients [7]. Current antiretroviral drugs are classified into the following categories: non-nucleoside reverse transcriptase inhibitors (NNRTIs), nucleoside/nucleotide reverse transcriptase inhibitors (NRTIs), protease inhibitors (PIs), integrase strand transfer inhibitors (INSTIs), fusion inhibitors, CCR5 antagonists, CD4 post-attachment inhibitors, and pharmacokinetic (PK) enhancers [8]. Despite their effectiveness in lowering viremia, current regimens lack the inclusion of an HIV-1 transcription inhibitor, thereby allowing for the continuous production of viral RNAs and failing to eliminate viral reservoirs [9,10,11]. Mechanistically, in viral transcription, the HIV-1 transactivator protein Tat interacts with the transactivation response element (TAR), a 59-nucleotide, non-coding stem-loop structure present in the 5′ LTR of the HIV-1 genome at the three-nucleotide UCU bulge, to facilitate the recruitment of transcription elongation factor b (P-TEFb) and induce the transcription of the integrated proviral genome [12,13,14,15]. Numerous studies have demonstrated that a bulge motif in TAR RNA is critical for its recognition by the Tat protein and have investigated the crystal structure of the TAR loop in complex with Tat/P-TEFb and found the interface interactions of TAR/Tat and TAR/Cyclin T1 [16,17,18,19,20]. Therefore, TAR RNA has been a target in antiviral research and the development of pharmaceuticals for a long time [21,22,23,24,25,26,27]. Our lab has previously shown the use of an ATP analog named CR8#13 as an effective inhibitor of Tat-activated transcription, which acts by decreasing the loading of Cdk9 onto the HIV-1 DNA [28]. Similarly, in another study, Tat peptide mimetic F07#13 was shown to decrease viral transcription by dissociating Cdk9 from its partner Cyclin T1 [29]. Didehydro-cortistatin A, the most promising HIV-1 transcription inhibitor to date, targets Tat protein by binding to the positively charged lysine of Tat’s TAR RNA-binding domain and reducing Tat’s binding affinity to TAR RNA [30]. Furthermore, the SWI/SNF complex, a chromatin remodeling complex, also plays a pivotal role in the regulation of Tat-activated transcription and allows RNA Polymerase II access to the HIV-1 proviral DNA [31,32].

In this study, we have screened a panel of small molecules that have been previously found to noncanonically bind the HIV-1 TAR element, a 5′ RNA element that is required for activated viral transcription [23]. We have identified five potential candidates that effectively inhibit viral transcription in myeloid and T cells without apparent toxicity. Interestingly, we observed that the infected cells of lymphoid and myeloid origin responded differently to the same TAR-binding molecules, which is consistent with previous studies [33,34]. Among the five candidates, mainly two molecules, 110FA and 102FA, showed inhibition of all viral protein expression. Additionally, a biotinylated RNA pulldown assay was performed to test the effect of shortlisted molecules on Tat–TAR RNA interaction, revealing that both molecules exhibit different mechanisms of action. 

Furthermore, a molecular dynamics (MD) simulation approach was used to determine the mechanism of action of 110FA in the presence of the P-TEFb complex, not just the TAR RNA alone. The complex dynamics elucidated how the TAR-binding molecule 110FA targets the Tat/TAR interface and prevents Tat binding to TAR RNA. Moreover, our in-silico analysis showed that 110FA conformers are in a pulled-out position from the RNA pocket compared to the TAR RNA alone, potentially inhibiting interaction between Cyclin T1 and the apical loop of the TAR RNA. This potentially makes 110FA a better inhibitor of viral transcription than a recently developed very potent TAR inhibitor named JB181 [26]. Furthermore, 110FA showed significant viral transcription inhibition in peripheral blood mono-nuclear cells infected with HIV-1 (98.6). In contrast, 102FA reduced viral transcription by disrupting the interaction between the Tat-associated Cyclin T1-Cdk9 subunit of the P-TEFb complex. We also investigated the recruitment of the SWI/SNF (switching-defective-sucrose non-fermenting) chromatin remodeling complex on HIV-1 LTR in the presence of TAR-binding molecules (110FA and 102FA). SWI/SNF, an important family of proteins recruited to the HIV-1 promoter, are human analogs of BAF and PBAF [32,35,36]. In chromatin remodeling and HIV transcriptional regulation, these complexes play opposing roles. BAF represses HIV transcription by positioning Nuc-1 downstream of the TSS of HIV, and aids in the establishment of latency, whereas PBAF acts as a co-factor for Tat transactivation. Our findings demonstrate that the TAR-binding molecules 110FA and 102FA can inhibit HIV-1 viral transcription by different mechanisms.

## 2. Results

### 2.1. Screening of TAR-Binding Molecules as Potential Transcription Inhibitors

**Figure 1:**
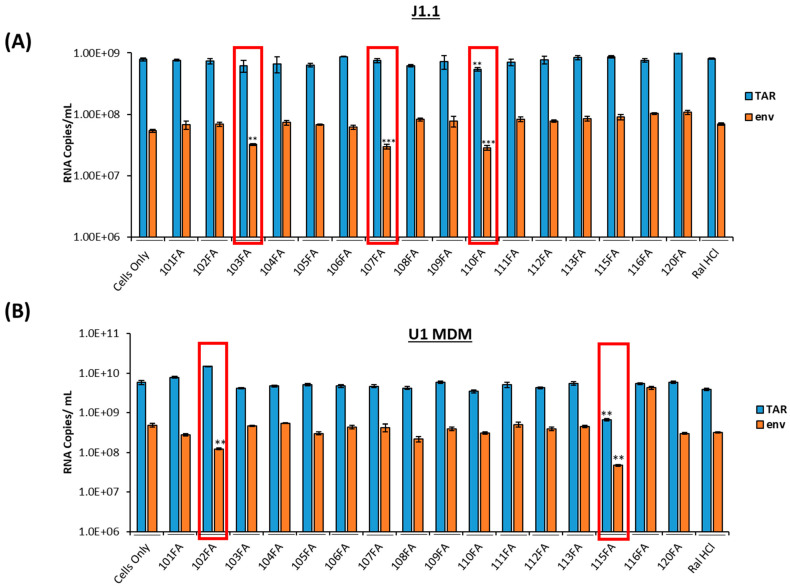
Screening of TAR-binding molecules to find out the potential transcription inhibitors in HIV-1-infected T cells and monocyte-derived macrophages (MDMs). (**A**) J1.1 cells (1 × 10^6^ cells/mL) were treated with 1 µM of TAR-binding molecules for 48 h. RNA was isolated and quantified using RT-qPCR with primers specific for HIV-1 TAR and genomic (*env*) RNA. (**B**) U1 cells were cultured and differentiated into MDMs (monocyte-derived macrophages) using 100nM phorbol 12-myristate 13-acetate (PMA) for five days. Differentiated MDMs (1 × 10^6^ cells/mL) were treated with 1 µM of TAR-binding molecules. After 48 h, RNA was isolated, and RNA samples were subjected to RT-qPCR with primers specific for HIV-1 TAR and genomic (*env*) RNA. A two-tailed Student’s *t*-test was used to assess significance in comparison to the untreated samples: ** *p* < 0.01; *** *p* < 0.001 and highlighted in red.

We have previously shown the use of ATP analogs and Cyclin T1/Cdk9 inhibitors to target HIV-1 transcription [28,29]. In this study, we utilized a panel of small molecules that have previously been shown to bind HIV-1 TAR RNA non-canonically [23] and screened for proviral transcription inhibition in two HIV-1-infected cell lines, J1.1 (T cells) and U1 monocyte-derived macrophages (U1 MDM), common host cell types infected by HIV-1. Since the J1.1 cell line is infected and highly productive, it is an excellent model for testing transcriptional inhibitors. U1 cells were cultured and differentiated into MDMs using 100 nM phorbol 12-myristate 13-acetate (PMA) for five days. Both cell types were treated with 1 µM of TAR-binding molecules for 48 h. RNA was isolated and quantified using RT-qPCR with primers specific for HIV-1 TAR and genomic (*env*) RNA from a previous study [37]. The RNA copy number was normalized to the RNA concentration input used in RT-PCR. The Student’s *t*-test was used to identify statistically significant downregulation of viral transcription in all experimental samples compared to a cell-only control. In our study, full-length transcripts (both unspliced and singly spliced) are represented by the *env* copies, which is also indicative of Tat-mediated transcription. In contrast, TAR RNA represents short abortive proviral transcripts and represents basal transcription. Our results indicated a statistically significant 2–5-fold decrease in TAR RNA copies (with 110FA only) and *env* copies with three molecules 103FA, 107FA, and 110FA in T cells, as shown in Figure 1A. Comparatively, in monocyte-derived macrophages (MDMs), as shown in Figure 1B, 115FA significantly reduced TAR and *env* RNA copies by 5–10-fold, respectively, whereas 102FA showed a significant 5-fold decrease in *env* RNA copies. The chemical structure of the molecules that were used to treat the cells is shown in Supplementary Figure S1. Collectively, both cell lines are responsive to different TAR-binding molecules. This discrepancy in the activity of the two molecules in infected T cells versus MDMs shows the potential for differences in HIV-1 proviral transcription among the two cell types, which is in line with the studies undertaken in the past [33,38,39,40]. Additionally, a consistent drop in *env* RNA copies with all five molecules indicates that these molecules potentially target the Tat-mediated transcription mechanism of viral RNA production.

### 2.2. Toxicity Studies of TAR-Binding Molecules 

**Figure 2:**
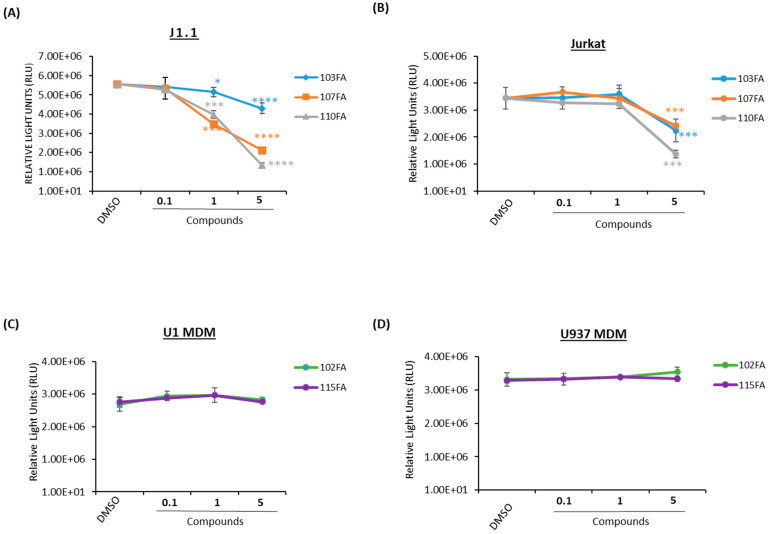
Dose titration of lead TAR-binding molecules in uninfected and HIV-1-infected cells. Fifty thousand cells were plated in a 96-well plate and treated with 0.1, 1, and 5 μM concentrations of TAR inhibitors or 0.1% DMSO, then allowed to incubate for 48 h prior to a CellTiter-Glo assay. (**A**) J1.1 (HIV-1-infected T lymphocytes) and Jurkat (uninfected T- lymphocytes) (**B**) cells were treated with 103FA, 107FA, and 110FA. (**C**) U1 MDMs and U937 MDMs (**D**) were treated with 102FA and 115FA. A two-tailed Student’s *t*-test compared untreated cells with treated cells. * *p* < 0.05; *** *p* < 0.001; **** *p* < 0.0001. Error bars, S.D.

After identifying potential transcription inhibitors, a series of titrations were performed in both T cells (J1.1, Jurkat) and macrophages (U1 and U937 MDMs) to determine the optimal dosage of the molecules to conduct further experiments. A CellTiter-Glo assay was utilized to analyze the viability of the TAR-binding molecule-treated cells. The results in Figure 2A show that infected T cells (J1.1) exhibited less cell viability at 1 μM concentration as compared to the uninfected T cells (Jurkat) in Figure 2B for all three shortlisted molecules (103FA, 107FA, and 110FA), whereas a significant decrease in cell viability was observed at 5 μM concentration in both cell types. In MDMs, 102FA and 115FA showed no reduction in cell viability at any of the doses in infected and uninfected cells (Figure 2C,D). Taken together, based on the viability results from uninfected T cells, a 1 μM concentration of TAR-binding molecules was chosen as the working concentration of the molecules for further assays.

### 2.3. Viral Protein Analysis of the Lead TAR-Binding Molecules in HIV-1-Infected Cells

Given that our results indicate a reduction in viral RNA, we hypothesized that viral transcription inhibition could also result in a decrease in viral protein expression in HIV-1-infected cells. J1.1 cells were treated with lead TAR-binding molecules 103FA, 107FA, 110FA, and U1 MDM cells with 102FA and 115FA at 1 µM concentrations each at 0 h and 48 h timepoints. After 72 h of treatment, whole cell lysates were isolated, run on gels, and probed with antibodies against HIV-1 viral proteins gp120, p24, and Nef. Additionally, β-actin was probed as a control.

**Figure 3:**
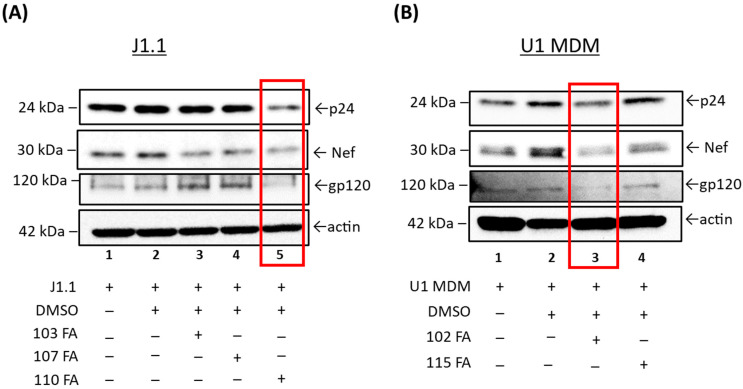
Western blot analysis of the lead TAR-binding molecules effects on HIV-1-infected T cells (J1.1) and U1 MDMs. (**A**) The three lead TAR-binding molecules (103FA, 107FA and 110FA) were used to treat J1.1 cells and two lead TAR-binding molecules (102FA and 115FA) were used to treat U1 MDM cells (**B**) at 1 µM each at 0 h and 48 h timepoints. After 72 h of treatment, whole cell lysates were generated, run on Western blots, and probed with antibodies against HIV-1 viral proteins p24, Nef and gp120. Additionally, β-Actin was probed as a loading control. Highest reduction in viral proteins expression is highlighted in red boxes.

Here, we found that two molecules, 103FA and 107FA (Figure 3A), reduced Nef expression levels by 28% and 14%, respectively (Lane 1 vs. Lanes 3, 4), without affecting the levels of the viral proteins p24 and gp120 in T cells. The third compound, 110FA, highlighted in red, reduced the expression of all viral proteins (Lane 2 vs. Lane 5), including p24, Nef, and gp120, by 38%, 36%, and 24%, respectively; densitometry data are shown in Supplementary Figure S2. In U1 MDMs (Figure 3B), a similar decrease in viral protein expression levels was observed with 102FA molecule (Lane 1 vs. Lane 3). Densitometry analysis in Supplementary Figure S3 shows 80% decrease in g120 and ~15% in p24 and Nef with 102FA. 115FA showed reduction in expression levels of gp120 only (Lane 1 vs. Lane 4). Collectively, TAR-binding molecules 110FA in T cells and 102FA in MDMs can inhibit the viral protein expression indicating that these molecules are potential inhibitors of Transcription elongation. Moreover, other molecules with no apparent reduction of viral protein expression might be inhibiting viral transcription by an unknown mechanism. Taken together, these observations suggested that the 110FA compound in T cells and 102FA in MDMs are most plausible HIV-1 inhibitors for viral transcription and translation.

### 2.4. Disruption of Tat–TAR RNA Interaction by Lead TAR-Binding Molecules

**Figure 4:**
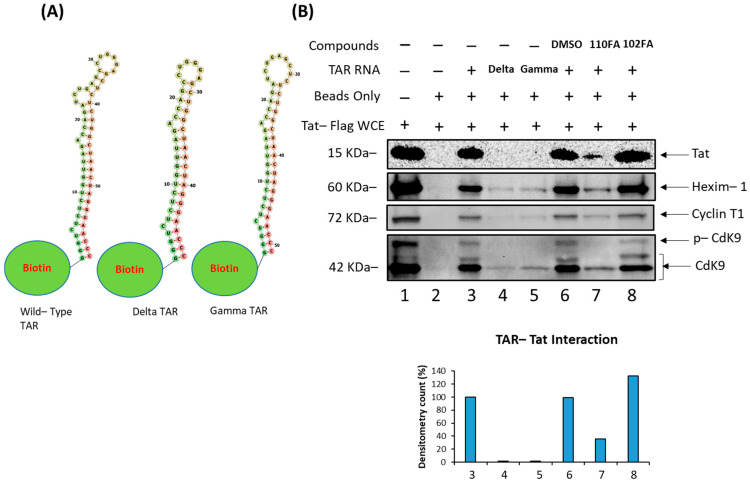
Disruption of Tat–TAR RNA interaction and change in RNA–protein interaction dynamics by 110FA and 102FA. (**A**) Structures of Wild-type (WT) TAR RNA, Delta TAR RNA (with Tat-binding bulge deleted), and Gamma TAR RNA (Cyclin T1 binding loop deleted) linked to the Biotin moiety. (**B**) Biotinylated RNA pulldown assay followed by immunoblot using protein lysate prepared from 293T cells expressing Flag-Tat and Biotin-WT TAR RNA or mutants coupled to beads. After incubation, the mixture was treated with 10 µM of the TAR RNA-binding molecules 110FA and 102FA. Coupled proteins were eluted from beads with Laemmli buffer, run on SDS-PAGE and detected with Anti-Flag, HEXIM-1, Cyclin T1, and Cdk9 antibodies.

We next asked whether the effect of leading TAR-binding molecules (110FA, 102FA) on the Tat–TAR RNA interaction could occur using a biotinylated pulldown assay (see Section 2.8). Briefly, Tat protein was extracted from the lysates of 293T cells that had been transfected with a Flag-Tat expression vector. A biotinylated 59-nt TAR RNA was immobilized on avidin-containing agarose beads. For the negative control, we used a 48-nt variant of the biotinylated TAR RNA that lacked the bulge region as a negative control (delta TAR RNA). We also included a second negative control, Gamma RNA, a 51-nt variant of TAR RNA with a missing Cyclin T1 binding loop. The secondary structures of RNAs attached to biotin molecules are shown in Figure 4A. Tat bound to the immobilized TAR RNA (Figure 4B, Lane 3), but not to the beads (Figure 4B, Lane 2), the immobilized delta TAR RNA, and gamma TAR RNA (Figure 4B, Lanes 3,4). The addition of 110FA (10 μM) to TAR RNA reduced Tat binding to the TAR RNA by 65% (Figure 4B, Lane 7). 102FA (10 μM) did not affect Tat binding to TAR RNA (Figure 4B, Lane 8), indicating that 102FA suppresses viral transcription or translation via some other mechanism. Furthermore, TAR RNA binding to HEXIM-1, Cyclin T1, and Cdk9 proteins was also examined in this assay; densitometry data are shown in Supplementary Figure S4. Additionally, to further validate the effect of 110FA in Figure 4B (Lane 7), a dose titration effect of 110FA was examined on the viral protein Tat and host proteins HEXIM-1, Cyclin T1, and cdk9 (Supplementary Figure S5). As expected, with an increase in the concentration of 110FA, a decrease in the recruitment of proteins occurred on Biotin-TAR RNA. Interestingly, HEXIM-1 binding to TAR RNA indicates the possibility of occupancy of an inactive HEXIM-1/P-TEFb complex onto TAR RNA (Lane 7) since the phosphorylated-Cdk9 (p-Cdk9) signal is almost lost with 110FA treatment, which is in line with a previous published report [41]. 

Collectively, 110FA interaction knocked out the Tat/P-TEFb complex away from TAR RNA. Moreover, binding of the inactive HEXIM-1/P-TEFb complex on TAR RNA points towards the existence of a fine interplay between the inactive HEXIM-1/P-TEFb and active Tat/P-TEFb complex on TAR RNA in the presence and absence of Tat inhibitors, respectively.

### 2.5. Computational Docking of TAR-Binding Molecules with TAR RNA

As only 110FA disrupted the interaction between Tat and TAR RNA, we next asked whether 102FA exhibits a different mechanism of transcription inhibition. The experimental rationale was that both 110FA and 102FA molecules decreased viral transcription and translation in T cells and U1 MDMs, respectively. Still, only 110FA could knock out Tat from TAR RNA in the biotin pulldown assay experiment. So, we performed molecular docking of 110FA and 102FA in the presence and absence of the P-TEFb complex with TAR RNA to mimic the intracellular environment using a molecular docking tool called HDOCK [42].

**Figure 5:**
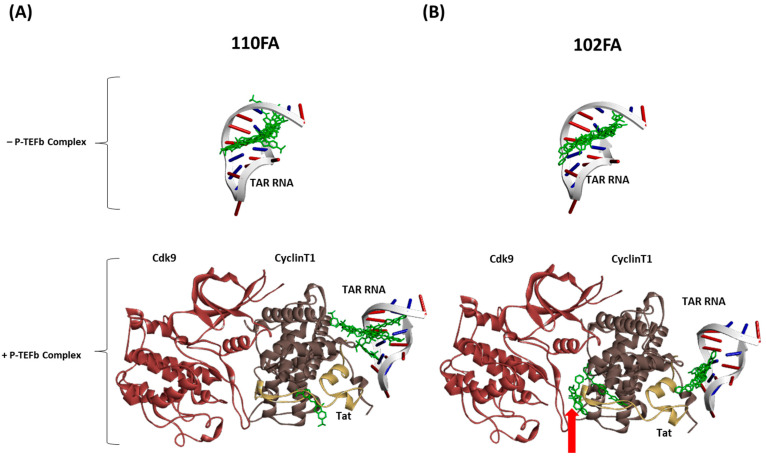
Computational docking of TAR-binding molecules with TAR RNA in the absence and presence of the P-TEFb complex using HDOCK. Molecular socking of the TAR-binding compound and the complex (PDB ID: 6CYT) was performed with HDOCKlite in the Linux (Ubuntu) system [31]. The molecular docking was performed separately with TAR RNA in the absence and presence of the P-TEFb/Tat complex with 110FA (**A**) and 102FA (**B**). Discovery Studio was employed as a visualization tool.

Our results in Figure 5 (top panels) showed that in the absence of the P-TEFb/Tat complex (TAR RNA alone), both 110FA and 102FA molecules bind stably in the TAR RNA pocket and interact with the Tat-binding bulge. However, in the presence of the P-TEFb/Tat complex, 110FA was stably bound in the RNA pocket (8 out of 10 configurations) (Figure 5A, bottom left panel). Moreover, 110FA molecules are in a “pulled-out” position from the RNA pocket compared to the TAR RNA alone (Figure 5A, top panel). This “pulled-out” position toward Cyclin T1, along with Tat knockdown, is potentially the cause of the transcription inhibition seen with 110FA. In contrast, with 102FA, in 50% configurations (5 out of 10), 102FA molecules appear segregated from the RNA pocket and are not pulled out towards Cyclin T1 (Figure 5B, bottom right panel). Moreover, in two configurations, 102FA intercalates between Cyclin T1-Cdk9 subunits (shown by red arrow). This could potentially explain why, in our biotinylation RNA pulldown experiment (Figure 4), 102FA did not remove Tat protein from TAR RNA yet reduced overall viral transcription and translation (Figure 1B and Figure 3B). Potentially, 102FA interrupts the interaction between the Tat-associated Cyclin T1/Cdk9 subunit of the P-TEFb complex and acts as a Cdk9 inhibitor. This is in line with the previously published studies showing Cdk9 inhibitors targeting HIV-1 transcription [28,29]. The binding energy of all the conformers (with and without complexes) for 102FA and 110FA is given in Supplementary Table S2.

### 2.6. Tat/Cyclin T1/Cdk9 Interaction in the Presence of TAR-Binding Molecules 

**Figure 6:**
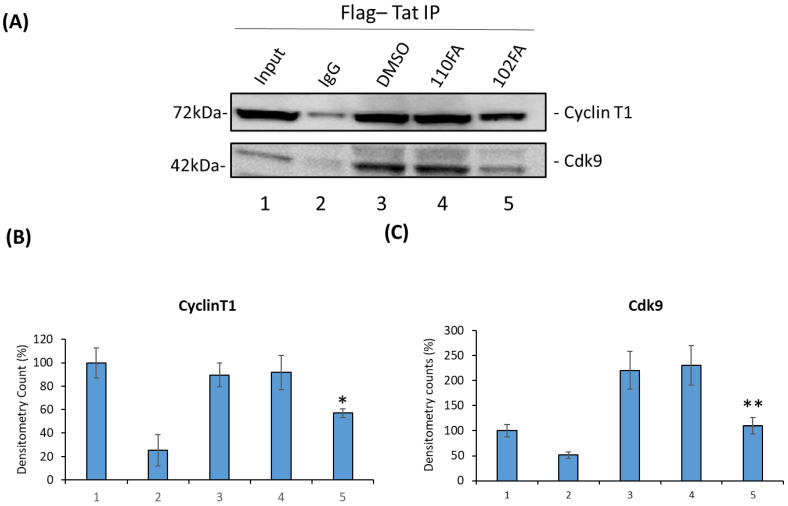
Effect of TAR-binding molecules on Tat-associated Cyclin T1 and Cdk9 protein levels. (**A**) HEK293T cells were transfected with the Flag-Tat plasmid (24 h) followed by TAR-binding molecules (110FA, 102FA) treatment at 1 μM concentration for 24 h, and cell lysate was collected. Cell lysates were incubated with protein A/G beads (pre-blocked with BSA), followed by immunoprecipitation with Anti-Flag antibody and IgG as a control overnight at 4 °C. Beads were then washed, Tat-associated proteins were eluted from beads using Laemmli buffer, and SDS-PAGE was run. Immunoblot was probed against the antibodies Cyclin T1 and Cdk9. Densitometry counts shown are results from three independent experiments with * *p* = 0.04 and ** *p* = 0.001 in comparison to the DMSO controls as shown in (**B**,**C**) for proteins Cyclin T1 and Cdk9, respectively.

We next attempted to validate the molecular docking results from Section 2.5 by conducting an immunoprecipitation assay. Briefly, HEK293T cells were transfected with the Flag-Tat plasmid (24 h) followed by TAR-binding molecules (110FA, 102FA) treatment at 1 μM concentration for 48 h, and cell lysate was collected. Next, cell lysates were incubated with protein A/G beads (pre-blocked with BSA), followed by immunoprecipitation with Anti-Flag antibody and IgG as a control overnight at 4 °C. The beads were then washed, Tat-associated proteins were eluted from the beads using Laemmli buffer, and they were run on SDS-PAGE. Immunoblots were probed against the antibodies Cyclin T1 and Cdk9. As expected, results in Figure 6A showed that 102FA has less impact on CyclinT1 association levels with Tat (decrease by 32%) as compared to DMSO control (Densitometry Data; Figure 6B), whereas it reduced the interaction of Cdk9 by more than 50% (Lane 3 vs. Lane 5), as shown in Figure 6C. However, 110FA showed no changes in comparison to DMSO control (Figure 6B,C; Lane 3 vs. Lane 4), indicating that 110FA does not decrease Tat-associated Cyclin T1 or Cdk9 levels. It is important to note that in the Tat/P-TEFb complex, Tat binds with the Cyclin T1 subunit of the P-TEFb complex [43] and maintains a steady phosphorylation state of Cdk9 via T-loop.

Overall, these results support the molecular docking results and explain how 102FA inhibits viral transcription or translation without removing Tat protein from TAR RNA in the cellular environment or in the P-TEFb complex setting. Thus, 102FA interferes with the interaction between Tat-associated Cyclin T1 and the Cdk9 subunit of P-TEFb. We also tested these molecules for their effect on normal cellular (not Tat-associated) levels of Cyclin T1/Cdk9 and did not observe a reduction in the expression levels of any of these proteins, revealing that TAR-binding molecules do not impact normal cellular transcription processes (Supplementary Figure S6). 

### 2.7. Alteration of SWI/SNF Complexes with TAR-Binding Molecules

**Figure 7:**
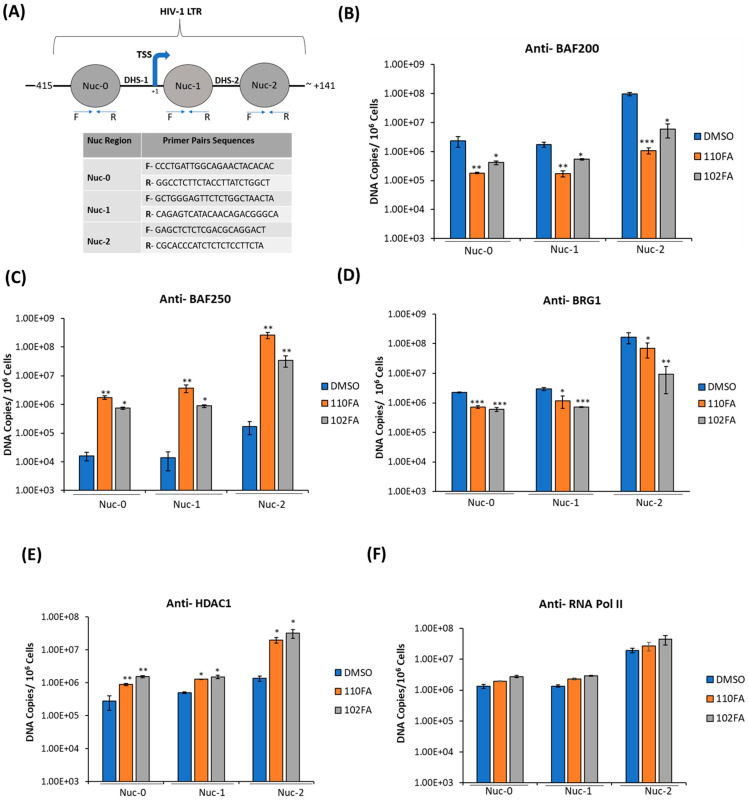

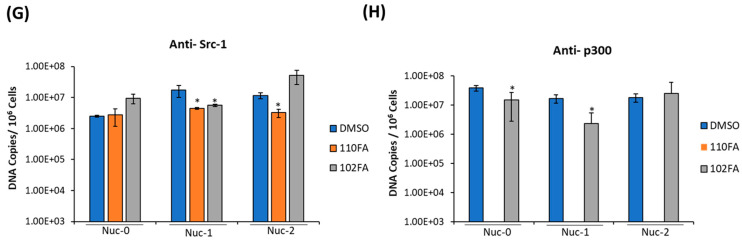
TAR-binding molecules alter transcription factor occupancy on the HIV-1 promoter. (**A**) Schematic representation of the positioning of nucleosomes at the HIV-1 integration site. (**B**–**G**) J1.1 cells were seeded to ~60% confluency and treated with 1 μM TAR-binding molecules (110FA, 102FA) or 0.01% DMSO for 72 h, followed by cross-linking of proteins to DNA using 1.0% formaldehyde. Chromatin was sonicated five times for 20 s each, generating DNA fragments of about 500–100 base pairs. Sonicated chromatin was subjected to immunoprecipitation with 10 μg of antibodies: BAF250, BAF200, BRG1, HDAC1, RNAP II, and Src-1. The immunoprecipitated DNA was extracted using the 1:1 phenol/chloroform method, purified by a PCR purification Kit (BiONEER), and amplified by quantitative PCR (qPCR). Primer pairs and conditions used for qPCR analysis are listed in Section 2.2. The absolute quantification of the samples was determined based on the cycle threshold value relative to the standard curve generated from serial dilutions of DNA from a CEM T-cell line containing a single copy of HIV-1 LAV provirus per cell (8 × 10^5^ cells). Two microliters per well of DNA were plated into a Master Mix (18 µL per well) containing Syber Green IQ Supermix (Bio-Rad, Hercules, CA, USA). Data are presented as DNA copies per 10^6^ cells for BAF200 (**B**), BAF250 (**C**), BRG1 (**D**), HDAC1 (**E**), RNA Pol II (**F**), Src-1 (**G**), and p300 (**H**). The average value of the IgG background for each primer set was subtracted from the raw data. A two-tailed Student’s *t*-test compared untreated (DMSO) with 110FA and 102FA treated. * *p* < 0.05; ** *p* < 0.01; *** *p* < 0.001. Error bars, S.D.

Next, we questioned whether the occupancy of transcription factors on HIV-1 LTR can be altered following treatment with TAR-binding molecules. To do so, J1.1 cells, which are infected with a wild-type LAI strain of the virus and show active transcription and viral shedding [44,45], were treated with 0.1% DMSO, 110FA, and 102FA (1 μM) followed by cross-linking of proteins (for 72 h) to DNA and used for the ChIP assay. To examine the recruitment of transcriptional factors, sonicated chromatin was subjected to immunoprecipitation with antibodies against suppressor SWI/SNF (BAF-specific BAF250), activator SWI/SNF (PBAF-specific BAF200), BRG1, HDAC1, RNA Pol II (RNAP II), and steroid hormone receptor coactivator 1 (Src-1). The immunoprecipitated DNA was analyzed by qPCR with primers specific for Nuc-0, Nuc-1, and Nuc-2. As shown in Figure 7B, BAF200 protein levels were significantly reduced at all three nucleosome positions in HIV-1 LTR, and a more significant drop was seen with 110FA (10–20-fold) in comparison to 102FA (5–16-fold). BAF200 is the integral subunit of PBAF, needed for transcriptional activation. On the contrary, BAF250 is BAF-complex-specific and acts as a transcriptional suppressor, showing increased levels at all three nucleosomes with a maximum of ~35 to 20-fold at Nuc-2 with 110FA and 102FA, respectively, as shown in Figure 7C, indicative of the suppression of transcriptional elongation. Similarly, BRG1, a core component of the SWI/SNF complex needed for Tat-mediated transactivation, showed less occupancy on nucleosomes (Figure 7D), with significantly lower levels at the Nuc-2 position (up to 10-fold with 120FA). Histone deacetylase (HDAC1) has previously been shown to maintain nucleosomes in a deacetylated state, which results in the inhibition of gene expression [46,47]. As expected, our results in Figure 7E showed elevated levels of HDAC1 at all nucleosome positions, with a maximum 14-to-23-fold increase with 110FA and 102FA, respectively. Since RNAP II is found on the promoters of actively transcribed genes, we next performed ChIP on RNAP II to confirm transcription inhibition in the promoter region. Interestingly, we noticed no significant change in RNAP II levels at all three nucleosome positions, as depicted in Figure 7F. This could possibly be due to paused RNAP II at the Nuc-0, Nuc-1, and Nuc-2 regions pointing towards a latent phenotype [48,49], which remains to be investigated. Moving forward, we also examined the occupancy of Src-1 in the LTR region. Src-1 is a nuclear cofactor involved in chromatin remodeling and promotes transcription through the recruitment of p300 and SWI/SNF [50,51]. Furthermore, viral Tat protein has been shown to cooperate with Src-1 and increase viral transactivation in the nucleus [52]. Our results in Figure 7G indicate an overall significant drop in Src-1 recruitment with 110FA at the Nuc-1 and Nuc-2 positions, whereas 102FA showed a significant decrease at the Nuc-2 position only. Next, we also examined the presence of p300 on HIV-1 LTR in the presence of TAR-binding molecules. p300, a histone acetyltransferase involved in chromatin remodeling and a transcriptional coactivator, interacts with the HIV-1 Tat protein and activates viral transcription [53,54,55]. The data in Figure 7H indicate that TAR-binding molecules decreased p300 recruitment in the LTR region. Interestingly, with 110FA molecules, the p300 levels were lower than the IgG control (the orange bar is not shown), which could be a major factor contributing to 110FA antiviral activity towards T cells over myeloid cells. Taken together, these results indicate that TAR-binding molecules result in a repressive chromatin environment characterized by less recruitment of PBAF (BAF200), BRG-1, Src-1, p300, a stalled RNAP II, and increased BAF250 and HDAC1 levels at the HIV-1 promoter.

### 2.8. Cell Viability and Transcription Inhibition in Primary Cells

**Figure 8:**
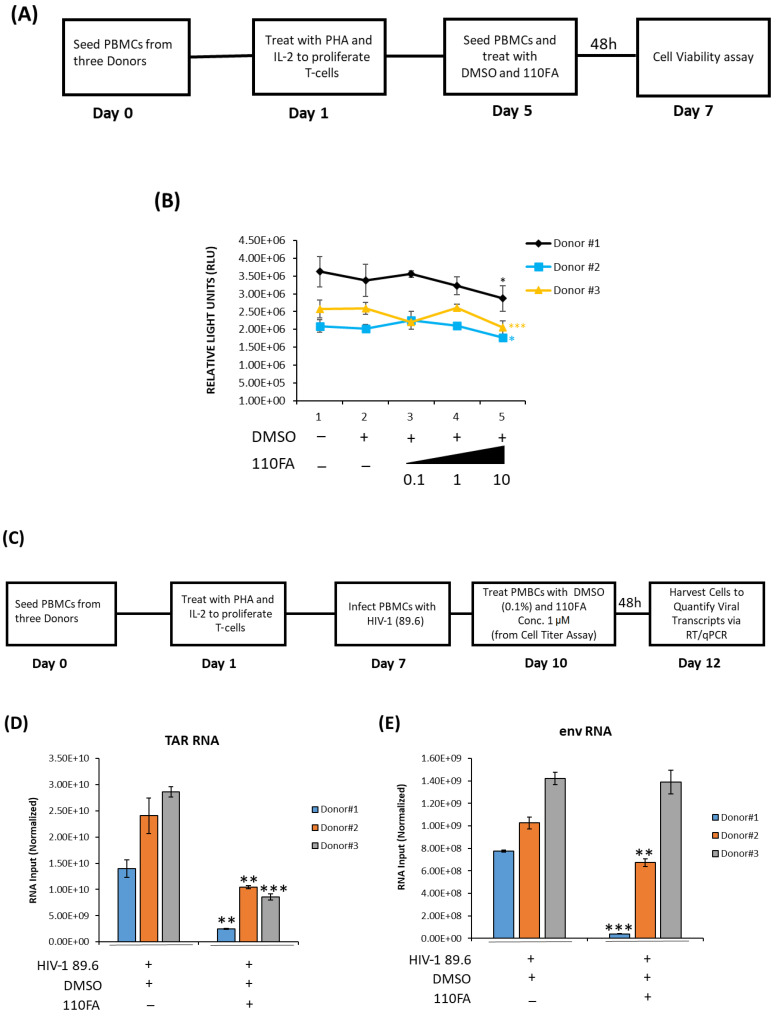
Cell viability and transcription inhibition in primary cells. (**A**) Diagram of the experimental design used for primary cell differentiation into T cells and cell viability assay. PBMCs from three donors were treated with phytohemagglutinin (PHA) and IL-2 and allowed to grow for five days to differentiate into T cells. Twenty thousand cells were seeded on day 5, followed by each PBMC treatment with 0.1, 1, and 10 μM concentrations of 110FA or DMSO, and incubated for 48 h for a CellTiter-Glo assay (**B**). (**C**) On day 7, cells were infected with HIV-1 (89.6). On day 10, cells were treated with a 1 μM concentration of the TAR inhibitor 110FA. After 48 h, RNA was isolated, and RNA samples were subjected to RT-qPCR with primers specific for HIV-1 TAR (**D**) and genomic (*env*) RNA (**E**). A two-tailed Student’s *t*-test was used to assess significance in comparison to the untreated samples: * *p* < 0.05; ** *p* < 0.01; *** *p* < 0.001.

To test whether the same trend of viral transcription inhibition can be observed in primary cells, PBMCs from three healthy donors were utilized for 110FA treatment. Briefly, as shown in Figure 8A, PBMCs from three donors were treated with phytohemagglutinin (PHA) and IL-2 and allowed to grow for five days to proliferate T cells. On day 5, each PBMC was treated with 0.1, 1, and 10 μM concentrations of lead TAR-binding compound 110FA or DMSO and incubated for 48 h for a CellTiter-Glo assay. The data in Figure 8B demonstrate that 110FA did not impact PBMC viability up to a dose of 1 μM for all three independent donors and observed a decrease in cell viability at a dose of 10 μM. Since no reduction in cell viability was seen at 1 μM concentration, we then used 1 μM concentration for further viral RT-qPCR assays.

Differentiated T cells were infected with HIV-1 dual tropic strain 89.6 on day 7, followed by treatment with a 1 μM concentration of 110FA as outlined in Figure 8C. TAR and *env* RNA copies were then measured after 48 h of treatment and normalized to the untreated control for each donor. Analysis of RNA copies in 110FA-treated cells demonstrates a statistically significant 89%, 57%, and 70% reduction in TAR RNA copies compared to untreated control in three donors, respectively (Figure 8D). Similarly, in Figure 8E, we observed a significant decrease in *env* RNA copies of 95% and 34% for the first two donors, and only a slight decrease of 2% for the third. Overall, a significant reduction in both TAR and *env* (full genomic RNA) transcripts suggests that 110FA inhibits Tat-activated transcription, potentially at multiple sites on the HIV-1 genome.

### 2.9. Molecular Simulation Derived Dynamical Calculations for the Tat–TAR RNA Interface in the Presence of the TAR RNA-Binding Molecule

Finally, we performed molecular dynamics (MD) simulations to investigate the Tat/TAR RNA interface dynamical changes as described in Section 2.5 As demonstrated in Figure 5B, docking simulations indicate that 102FA predominantly targets the CDK9–Tat interface and does not interact with TAR RNA. This computational result validated the results from biotinylated TAR pulling-down assay in Figure 4B, where similar patterns in Lanes 6 and 8, in the absence and presence of 102FA, respectively, demonstrate that 102FA has no impact on RNA–protein interactions, specifically those between TAR and CDK9/Cyclin T1/Tat. Therefore, 102FA is excluded from the analysis of the dynamical properties of the RNA–protein interface in this section. Therefore, steered molecular dynamics (SMD) simulations were performed to investigate the Tat–TAR-dependent interaction mechanism with and without the TAR-binding molecule 110FA. 

**Figure 9:**
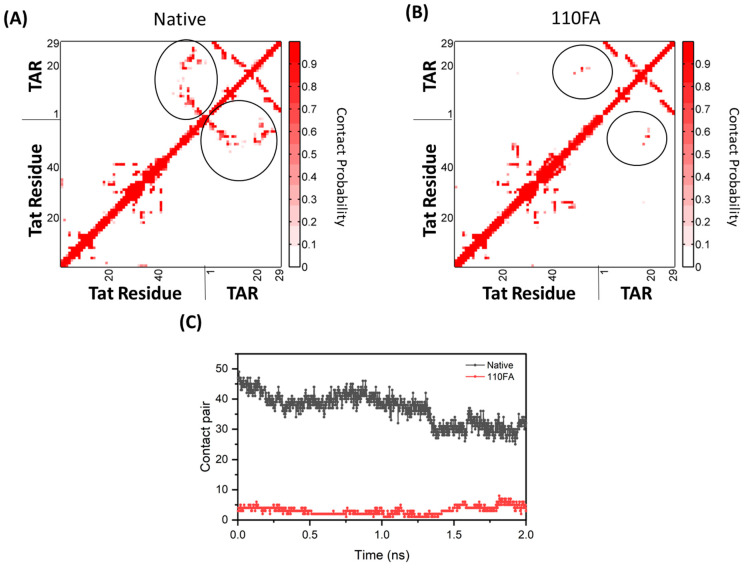
Molecular simulation-derived dynamical change calculations between Tat–TAR RNA complex with and without the TAR-binding molecule (circled in black). The interaction analysis between Tat (ARM, I40–R52) and TAR RNA upon 110FA binding. (**A**) The contact probability of Tat and TAR in the native state; (**B**) 110FA-bound state simulation; (**C**) contact pairs between Tat–TAR RNA in the native and 110FA-bound states.

Our SMD results showed that in the absence of the TAR-binding molecule (110FA) in native conformation (Figure 9A), the contact probability between Tat residues and TAR RNA is high (0.8), as indicated by the red grid. In contrast, with 110FA (Figure 9B), the contact probability is decreased by 75% (0.2). This is further supported by contact map calculations, where the interaction between Tat and TAR RNA is represented as contact pairs. As per previous findings, two residues are considered to be in contact when any two heavy atoms of a pair of residues are closer than 4.5 Å [56,57]. In native confirmation (absence of 110FA), the contact pairs between TAR RNA and Tat were close to 50, reduced to 5 upon 110FA binding (Figure 9C). To ensure the accuracy of the contact map distance chosen (4.5 Å), contact map analysis was also conducted at different cut-off values (4 and 5 Å), as shown in Supplementary Figure S7, and no difference was observed in contact pairs. The contact pairs in native and 110FA-bound states are shown in Supplementary Table S1. Taken together, the steered simulation studies show that 110FA stably binds in the TAR RNA pocket and prevents Tat binding to TAR RNA by keeping the Tat/P-TEFb complex away from TAR RNA, as depicted in Supplementary Figure S8. 

## 3. Discussion

We and others have previously demonstrated that, despite effective viral suppression by cART, viral reservoirs, including those in the CNS, are transcriptionally active, resulting in the synthesis of short, non-coding, and occasionally full-length genomic RNAs via non-processive and basal transcription, respectively. Several studies have shown approximately 1 × 10^3^ copies of cell-associated RNA in both circulating CD4+ T cells [9,10] and myeloid cells from various brain areas [58]. Therefore, it is necessary to include viral transcription inhibitors in current regimens targeting HIV-1 proviral transcription to achieve complete viral shutdown. At the transcriptional regulation level, HIV-1 transcription is controlled by the interaction between the viral Tat protein and the trans-activator response element (TAR) RNA [59]. The TAR stem-loop structure has a crucial component: a three-nucleotide bulge UCU (positions 23–25) directly targeted by Tat [60,61]. Novel antiviral drugs targeting the Tat–TAR complex are proving appealing targets for researchers. 

Our findings demonstrate that the TAR-binding molecules 110FA and 102FA can inhibit HIV-1 viral transcription by different mechanisms. In this study, we screened a series of TAR-binding molecules that have been previously found to bind HIV-1 transactivation response (TAR) elements non-canonically [23], and we shortlisted five molecules (Figure 1) that were further analyzed to identify the most effective inhibitor and gain insight into their mechanism of action. Another significant finding from this study was the discovery of differences in the responses of infected T cells and myeloid cells to the same molecules (Figure 1). This is not the first time that T cells or myeloid cells have shown distinct pharmacological responses. For instance, HDAC inhibitors activate latent provirus in dormant CD4+ T cells, whereas they inhibit viral production in macrophages via autophagy activation [34,62]. Likewise, in another study, mycophenolate reduced *env* RNA copies in HIV-1-infected J1.1 (T cells) while increasing *env* copies in promyelocytic OM10.1 cells [33]. Our lead compound, 110FA, inhibited Tat-mediated HIV-1 transcription (HIV-1 TAR and *env* RNA production) and reduced the expression of viral proteins gp120, p24, and Nef in T cells (Figure 2A). On the contrary, in monocyte-derived macrophages (MDMs), 102FA showed similar effects (Figure 2B), except for the fact that it was not a Tat-mediated mechanism, as shown by the biotinylated RNA pulldown assay (Figure 4), since there was no reduction in Tat occupancy on TAR RNA. One study has shown that HEXIM-1 can bind to TAR RNA [41]. Interestingly, HEXIM-1 binding to TAR RNA in Figure 4; Lane 7 indicates the possibility of occupancy of an inactive HEXIM-1/P-TEFb complex onto TAR since the phos-phorylated-Cdk9 (p-Cdk9) signal is almost lost with 110FA treatment. This finding points towards the existence of a fine interplay between inactive HEXIM-1/P-TEFb and active Tat/P-TEFb complex on TAR RNA in the presence and absence of Tat inhibitors, respectively. The HEXIM-1/TAR interaction suggests an interesting mechanism of occupancy to look at for future studies. 

Our lab and others have previously shown that Cdk9 inhibitors can target HIV-1 transcription. Here, we hypothesized that 102FA might reduce viral transcription by interfering with the Cyclin T1–Cdk9 interaction. In recent years, computational docking has been widely used to study the interactions between TAR RNA and various ligands [63,64,65]. Therefore, to solve the puzzle, a molecular modeling approach was used (Figure 5), and indeed, 102FA intercalates between the Cdk9 and the Cyclin T1 subunit in two different conformations, which was further validated by an immunoprecipitation assay (Figure 6). Further confirmation of these predictions is needed by either NMR or crystallography analysis in future work. The rationale for why only 110FA interaction with TAR RNA is altered upon addition of proteins is unclear given the similarity of 102FA and 110FA structures. Next, we also investigated the recruitment of the SWI/SNF to the HIV-1 promoter to figure out the ongoing dynamics between chromatin remodeling and HIV transcriptional regulation. Overall, we have shown that TAR-binding molecules result in repressive chromatin environment at nucleosomes Nuc-0, Nuc-1, and Nuc-2 (Figure 7). The stalled RNA polymerase II recruitment in the nucleosome region (Nuc-0, Nuc-1, and Nuc-2) further supported the findings. Whether stalled RNA Pol II indicates the development of a latent phenotype remains to be investigated. 

One of the recent studies showed that JB181, an extremely potent inhibitor (picomolar; pM affinity) of Tat binding to TAR RNA, weakly inhibits loading of the SEC core (P-TEFb, AFF4, and Tat) on TAR in vitro and permits the interaction of Cyclin T1 with the TAR apical loop [26], suggesting the need to develop molecules that can simultaneously inhibit both Tat as well as Cyclin T1 interaction with TAR RNA. Along these lines, our molecular docking results (Figure 5A; bottom panel) showed that the 110FA molecules are in a pulled-out position from the RNA pocket compared to the TAR RNA alone (Figure 5, top panel). This “pulled-out” position toward Cyclin T1, along with Tat knockdown, is potentially the cause of the transcription inhibition seen with 110FA. In contrast, potent JB181 binds deep into the TAR RNA pocket and does not interact with Cyclin T1, thus weakly inhibiting viral transcription. In other words, “true” RNA ligands like JB181 (interacts with TAR RNA only) show a weak effect on transcription regulation. In contrast, 110FA, an RNA-Protein ligand (interacting with TAR RNA and Cyclin T1 protein), reduces viral transcription significantly. On the other hand, 102FA, a potential Cyclin T1-Cdk9 inhibitor in complement with 110FA, might be a feasible strategy to achieve complete HIV-1 transcription inhibition in cells of monocytic origin. Tat-mediated transcription inhibition by 110FA was further evaluated by molecular simulation dynamic calculations (Figure 9), and the 110FA molecule restricted Tat binding to TAR RNA by 75%. Using primary cells from three donors, we demonstrated the ability of 110FA to inhibit viral transcripts (TAR and *env*) (Figure 8) without toxicity at a 1 μM concentration.

In summary, molecules 110FA and 102FA identified in this study successfully inhibited HIV-1 transcription by two different mechanisms. Additionally, shifting of the TAR RNA loop towards Cyclin T1 upon molecule binding during molecular simulation studies suggested that targeting the TAR loop and Tat-binding UCU bulge together should be an essential feature of TAR-binding molecules/inhibitors to achieve complete viral transcription inhibition. Moreover, the findings of this study necessitate further investigation of the fundamental transcriptional and viral production differences between these two major HIV-1 provirus reservoir hosts. Additionally, because these two infected cell types respond differently to the same molecules, developing unique therapeutic interventions for each will almost certainly be required to elicit functional or sterilizing elimination of the provirus from these two cell lineages.

## 4. Materials and Methods

### 4.1. Cell Culture and Reagents

Jurkat (uninfected T lymphocytes), J1.1 (HIV-1-infected T lymphocytes), U1 (HIV-1-infected promonocytic), and U937 (promonocytic) cells were cultured in complete RPMI 1640 media with 10% fetal bovine serum (FBS), 1% L-glutamine, and 1% penicillin/streptomycin (Quality Biological, Gaithersburg, MD, USA) and incubated in 5% CO_2_ at 37 °C. The cells mentioned above were provided by the National Institutes of Health’s (NIH) AIDS Reagent program. Phorbol 12-Myristate 13-Acetate (PMA; 100 nM and CAT: 16561-29-8, Cayman Chemical, Ann Arbor, MI, USA) was used to differentiate monocytes into monocyte-derived macrophages (MDMs) for 5 days. Cells were treated with TAR-binding molecules, which have been previously described in Abulwerdi et al. [23] and are shown in Supplementary Figure S1. HEK293T cells (CRL-3216, ATCC, Manassas, VA, USA) were cultured in DMEM (Dulbecco’s Modified Eagle’s Medium) media.

A set of primary PBMCs (Precision For Medicine, Frederick, MD, USA) were cultured in vitro first in PHA/IL-2 (phytohaemagglutinin/Interleukin-2) for 5 days to obtain activated T cells. On day 5, cells were harvested for the drug-titer assay. On day 7, T cells were then infected with the HIV-1 89.6 strain (MOI: 10) for 3 days, and on day 10, T-cell cultures were then treated with 110FA for 48 h. On day 12, cells were processed for RNA analysis. 

### 4.2. RNA Isolation, Creation of cDNA, and Quantitative Real-Time PCR (RT-qPCR)

For the isolation of total RNA, cells were harvested, washed once in 1× PBS without calcium or magnesium, and resuspended in 50 µL of 1× PBS. Total RNA was isolated from cell pellets using Trizol Reagent (Invitrogen, Waltham, CA, USA) as described by the manufacturer’s protocol. cDNA was generated using GoScript Reverse Transcription Systems (Promega, Madison, WI, USA) using Envelope Reverse (5′-TGG GAT AAG GGT CTG AAA CG-3′; Tm = 58 °C) and TAR Reverse (5′-CAA CAG ACG GGC ACA CAC TAC-3′, Tm = 58 °C) primers. Serial dilutions of DNA from a CEM T-cell line containing a single copy of HIV-1 LAV provirus per cell (8 × 10^5^ cells) were used as the quantitative standards. cDNA samples (2 µL per well) were plated into a Master Mix (18 µL per well) containing IQ Supermix (Bio-Rad), TAR Forward Primer (5′-GGT CTC TCT GGT TAG ACC AGA TCT G-3′), TAR Reverse Primer (5′-CAA CAG ACG GGC ACA CAC TAC-3′), and TAR Probe (5′56-FAM-AG CCT CAA TAA AGC TTG CCT TGA GTG CTT C-36-TAMSp-3′). The qPCR conditions were as follows: one cycle for 2 min at 95 °C, followed by 41 cycles of 95 °C for 15 s and 58 °C for 40 s. Reactions were performed in triplicate using the BioRad CFX96 Real-Time System. Quantitation was determined using cycle threshold (Ct) values relative to the 8 × 10^5^ standard curve using the BioRad CFX Manager software (version 3.0). The primer pairs and cycle conditions used for qPCR to quantify DNA copies at the nucleosome position are adopted from Li, C. et al. [66] and given below:

Nuc-0-F CCCTGATTGGCAGAACTACACAC

Nuc-0-R GGCCTCTTCTACCTTATCTGGCT

Nuc-1-F GCTGGGAGTTCTCTGGCTAACTA

Nuc-1-R CAGAGTCATACAACAGACGGGCA

Nuc-2-F GAGCTCTCTCGACGCAGGACT

Nuc-2-R CGCACCCATCTCTCTCCTTCTA

The qPCR steps were as follows: one cycle for 2 min at 95 °C, followed by 41 cycles of 95 °C for 15 s, 66 °C for 40 s, and 72 °C for 30 s. All reactions were run in triplicate on the CFX96 Real-Time PCR Detection System (Bio-Rad, Hercules, CA, USA), and the generated raw data was analyzed using Microsoft Excel 2016.

### 4.3. Cell Viability

Cell viability was assessed by plating 5 × 10^4^ cells or 2 × 10^4^ PBMC cells and treating them with TAR RNA-binding molecules (grown in fresh RPMI media and supplemented as described above) into a 96-well cell culture plate. After 2 days, cell viability was tested using the CellTiter-Glo Luminescent Cell Viability Assay (Promega). Luminescence was measured using the GloMax Multi-Detection System (Promega). All cell viability assays were conducted in biological triplicate, and the background signal was normalized with fresh RPMI media.

### 4.4. Preparation of Whole Cell Extracts 

Uninfected/infected T lymphocytes and macrophages were centrifuged at 1800 rpm at room temperature for 5 min. Cell pellets were washed with 1× Phosphate Buffer Saline (PBS) without Ca++ and Mg++ and resuspended in lysis buffer comprising 120 mM NaCl, 50 mM Tris-HCl, pH 7.5, 50 mM NaF, 5 mM EDTA, 0.5% NP-40 (NP40), 0.2 mM Na_3_VO_4_, 1 mM DTT, and 1 complete protease inhibitor cocktail tablet/50 mL (Roche Applied Science, Mannheim, Germany). Cell pellets were incubated on ice for 20 min, with vortexing every 5 min. The whole cell lysate was separated from cell debris by centrifugation at 10,000 rpm at 4 °C for 10 min. The Bradford assay (Bio-Rad) was used to determine the protein concentration according to the manufacturer’s protocol.

### 4.5. Western Blot Analysis and Antibodies

Laemmli buffer (Tris-glycine-SDS buffer and β-mercaptoethanol) was added to whole cell lysate samples. Samples were heated at 95 °C for 3 min, then loaded into 4–20% Tris-glycine gels (Invitrogen) with a Precision Plus Protein™ Standard (BioRad) and fractionated at 150 V. Gels were transferred onto Immobilon polyvinylidene fluoride (PVDF) membranes (Millipore, Burlington, MA, USA) at 50 milliamps overnight. Five percent milk in PBS containing 0.1% Tween-20 (PBS-T) was used to block the protein membranes for 2 h at 4 °C. Primary antibody in PBS-T was added to the membranes prior to overnight incubation at 4 °C; primary antibodies include: α-Flag M2 (Cat: F1804; Sigma Aldrich, St. Louis, MO, USA, 1:1000), α-HEXIM1 (Cat: 12604; Cell Signaling Technology, Danvers, MA, USA, 1:1000), α-Cyclin T1 (Cat: 81464, Cell signaling Technology, 1:1000), α-Cdk9 (Cat: 2316, Cell signaling Technology, 1:1000) and β-actin (Cat: ab-49900; Abcam, Waltham, MA, USA, 1:5000), α-p-Cdk9 (T-186) (Cat: ab79178, Abcam, 1:1000), gp120, α-p24 and α-Nef were obtained from NIH AIDS Reagent Program, Manassas, VA, USA. Membranes were washed three times with PBS-T (5 min/wash). Complementary HRP-conjugated secondary antibodies were added, followed by incubation for 2 h at 4 °C. Membranes were washed twice with PBS-T and once with PBS, 5 min per wash. HRP luminescence was activated with Clarity Western ECL Substrate (Bio-Rad). Membranes were developed using the Molecular Imager ChemiDoc Touch system (Bio-Rad). 

### 4.6. Prediction of RNA Secondary Structure

For prediction of the RNA secondary structures, the sequence of HIV-1 Wild-type TAR (59 nucleotides, 5′-GGGUCUCUCUGGUUAGACCAGACUGAGCCUGGGAGCU-CUCUGGCUAACUAGGGAACCC-3′), mutant delta TAR with the deletions of Tat-binding bulge nucleotides 21–27 and 38–41 (48 nucleotides, 5′-GGGUCUCUCUGGUUAGACCAGCCUGGGAGCUGGCUAACUAGGGAACCC-3′), and gamma TAR with the deletions of Cyclin T1 binding nucleotides 29–36 (51 nucleotides, 5′-GGGUCUCUCUGGUUAGACCAGACUGAGCU CUCUGGCUAACUAGGGAACCC-3′) were submitted to the Vienna RNA secondary structure server. The server predicts the minimum free energy (mfe) secondary structures for single RNA sequences/DNA sequences. The MFE structure of an RNA sequence is the secondary structure that contributes a minimum of free energy. This structure is predicted using a loop-based energy model and the dynamic programming algorithm introduced by Zuker et al. [67]. This server also calculates the full equilibrium partition function for secondary structures and the probabilities of various substructures by using the partition function (pf) algorithm proposed by McCaskill [42]. All the secondary structure predictions were performed at a temperature of 37 °C, keeping all the other parameters to default. 

### 4.7. Transfection

The Cell-Porator™ [Life Technologies, Inc.; Bethesda Research Labs (BRL), Frederick, MD, USA] was used to transfect HEK293T cells per the manufacturer’s instructions. Briefly, HEK293T cells (5 × 10^6^ cells per sample) were electroporated in DMEM media containing 10% FBS and 5% L-glutamine. The cell lines were transfected with DNA (20 µg) at the following parameters: a capacitance of 800 µF, low resistance, a pulse voltage of 230 V, and a fast charge rate.

### 4.8. Biotinylation Pulldown

Biotin-HIV-1 Wild-type TAR (59 nucleotides, 5′GGGUCUCUCUGGUUAGACCAGACUGAGCCUGGGAGCU-CUCUGGCUAAC UAGGGAACCC-3′), Biotin-mutant deltaTAR (48 nucleotides, 5′-GGGUCUCUCUGGUUAGACCAGCCUGGGAGCUGGCUAACUAGGGAACCC-3′) were generous gifts from Dr. Sergei Nekhai (Howard University, Washington, DC, USA) and gamma TAR (51 nucleotides, 5′-GGGUCUCUCUGGUUAGACCAGACUGAGCUCUCUGGCUAACUAGGGAACCC-3′) was designed and ordered from IDT technologies (Coralville, IA, USA) with Biotin attachment. HEK293T cells were co-transfected with the Flag-Tat expression vector (a generous gift from Dr. Zachary Klase, Drexel University, Philadelphia, PA, USA) for 48 h before lysis in the whole cell lysate buffer (50 mM Tris-HCl, pH 7.5, 0.5 M NaCl, 1% NP-40, 0.1% SDS, protease inhibitor). Streptavidin-agarose beads were incubated with BSA, and tRNA was added for blocking. Blocked beads were then incubated with either 10 µg WT TAR RNA, 10 µg delta-TAR RNA, or 10 µg gamma TAR RNA for 4 h at 4 °C. Beads bound with Biotin-RNA were incubated with 200 µg of whole cell extract in TAK buffer (50 mM Tris-HCl, pH 8.0, 5 mM MgCl_2_, 5 mM MnCl_2_, 10 M ZnSO_4_, 1 mM DTT, 1 mM DTT). Next, we added 10 µM of 110FA to each tube for 8 h. The proteins bound to the beads were eluted in 1X SDS loading buffer after centrifugation for 5 min at 1000× *g*. It was then transferred to a PVDF membrane, where it was immunoblotted with antibodies, and chemiluminescence detection was carried out as described previously in Section 2.5.

### 4.9. ChIP Assay

Chromatin immunoprecipitation (ChIP) assays were performed on cells using antibodies as described below. Cells were seeded to ~60% confluency, treated with TAR-binding molecules or DMSO, then processed for ChIP, beginning with cross-linking proteins to DNA with 1.0% formaldehyde. Chromatin was sonicated five times for 20 s each, generating DNA fragments of about 500–100 base pairs. The sonicated supernatants containing the DNA were diluted with ChIP dilution buffer (0.01% SDS, 1% Triton X-100, 1.2 mM EDTA, 16.7 mM Tris–HCl pH 8.1, and 167 mM NaCl) to a total volume of 5.5 mL and precleared by rotating for 1 h at 4 °C with ChIP-prepared protein A/G beads (beads were washed twice with 1 mL TNE50 + NP-40, resuspend in 650 μL; add 40 μL of ssDNA (10 mg/mL) and 75 μL BSA (10 mg/mL)). No proteases or RNAses were used for the extraction. The extract was centrifuged at 3000 rpm for 10 min at 4 °C, and the lysate was transferred to a fresh tube. Supernatant (500 μL) was reserved for input, and then 10 μg of each antibody α-RNAP II (Cat: sc-899; Santa Cruz Biotechnology, Santa Cruz, CA, USA), α-BRGl (Cat: sc-10768; Santa Cruz Biotechnology, CA, USA), α-BAF200 (Cat: sc-81050; Santa Cruz Biotechnology, CA, USA), α-BAF250 (Cat: sc-48791; Santa Cruz Biotechnology, CA, USA), α-HDACl (Cat: c-7872; Santa Cruz Biotechnology, CA, USA), α-p300 (Cat: sc-585; Santa Cruz Biotechnology, CA, USA) and α-Src-1 (Cat sc-995; Santa cruz Biotechnology were added to the reaction mixture. After overnight rotation at 4 °C, the immune complexes were collected by adding ChIP-prepared protein A/G beads. After extensive washes, the immune complexes were treated with proteinase K (30 min, 37 °C) and then eluted with a 1% SDS/NaHCO_3_ solution for 30 min at room temperature. The eluted complexes were treated with an NaCl solution and reverse cross-linked overnight. DNA was extracted using 1:1 phenol/chloroform (500 μL), followed by the addition of 1 mL of absolute ethanol and 3 M sodium acetate (50 μL), and incubation at −20 °C for at least 20–30 min. The solution was spun for 20 min at 14,000 rpm at 4 °C, followed by a 70% ethanol wash and a 5 min spin. DNA pellet was resuspended in 1× TE and stored at 4 °C. Afterwards, DNA was purified by the PCR purification Kit (BiONEER; Oakland, CA, USA) and amplified by PCR. For qPCR, enriched DNA was also quantified using an ABI Prism 7100 instrument and SYBR green (Applied Biosystems, Foster City, CA, USA). The average value of the IgG background for each primer was subtracted from the raw data. Primer pairs and the method used for quantitative PCR analysis are listed in Section 2.2.

### 4.10. Immunoprecipitation Assay

HEK293T cells were transfected as described above (Section 2.7) for 24 h, followed by DMSO (0.1%), 110FA, or 102FA (1 µM) treatment for an additional 24 h. Cells were lysed in a whole cell lysate buffer supplemented with protease inhibitors. The Bradford assay (Bio-Rad) was used to determine the protein concentration according to the manufacturer’s protocol. Protein A/G agarose beads were blocked with 5% Bovine Serum Albumin (Sana Cruz Biotechnology, Santa Cruz, CA, USA) (BSA) for 2 h. Blocked beads were incubated with the α-Flag M2 antibody for 4h at 4 °C before being combined with protein lysate overnight. The beads were washed three times in 500 µL of PBS-T buffer and heated for 5 min at 95 °C. Eluted proteins were Western blotted for the presence of Cyclin T1 and Cdk9 proteins.

### 4.11. Molecular Docking

The PDB file for the potential TAR-binding molecules was derived from PubChem [68], and the PDB files for the TAR RNA and protein complex (PDB ID: 6CYT) were taken from the RCSB PDB database [69]. Molecular docking was performed with HDOCKlite in the Linux (Ubuntu) system [19,42]. HDOCK is a docking program that is developed for nucleic acid docking. It contains the global sample putative binding modes that use an improved shape-based pairwise scoring function. The two ligands (110FA and 102FA) were docked separately with the complex crystal structure via template-based docking, and the top 10 conformations with the highest scores were kept for further analysis. In addition, the Discovery Studio software (https://discover.3ds.com/discovery-studio-visualizer-download) package from BIOVIA (San Diego, CA, USA) was employed for visualization. 

### 4.12. Molecular Dynamics (MD) Simulations 

The initial structure of the P-TEFb complex in the molecular dynamics (MD) simulations was derived from the crystal structure (PDB: 6CYT) [19]. The extended Tat is built with the software Chimera-1.13.1 according to the NMR crystal (PDB: 6MCE) [20,70]. The AMBER module Antechamber was employed to calculate the atom charge and optimize the structure. AMBER20 was adapted to process the complex structure to remove the initial solvent and ions [71]. Then, the complex was solvated in a cubic box with periodic boundary conditions. The ions were added to neutralize the net charge of the system. The particle mesh Ewald (PME) method was used to calculate the long-range electrostatic interactions [72]. The energy minimization was performed with the steepest descent method until the maximal iteration steps reached 20,000. Next, the system was heated to 300 K in two steps in the NVT. Subsequently, the NPT ensemble was divided into four steps to relax the atom restrictions progressively. The hydrogen bonds were constrained by the SHAKE algorithm [73]. 

In the steered molecular dynamics (SMD) simulations, the trajectory was partitioned into five equal segments and 25 trajectories per stage. TAR RNA was pulled away from the binding interface of Cyclin T1 with a 10 Å/ns pulling speed. The spring constant (k) was set at 7.2 kcal mol^−1^ Å^−2^, according to Ozer et al. [74]. The steered force was constant in the steered MD simulations. The snapshots were saved every 1000 steps as they determined the distance and force information. Finally, a 50 ns production simulation was performed in the SMD, and 3.2 ns of the optimal pathway was calculated with a python script to perform analysis.

### 4.13. Densitometry Analysis

Densitometry analysis was performed using ImageJ software (https://imagej.net/). Densitometry data were normalized using a two-step process to control both exposure and loading. First, background measurements for each membrane were subtracted from the measurement of interest. Next, each protein band was normalized to the corresponding actin. Normalized counts are represented as an increase or decrease relative to the untreated control (lane 1 is set to 100%). Reduction trends were calculated by subtracting the normalized treated lane counts from the normalized control lane counts.

### 4.14. Statistical Analysis

Standard deviations (SD) were used to assess sample variance in all quantitative experiments via Microsoft Excel. A Student’s *t*-test was used to assess statistical significance or *p*-values. *p*-values were considered * significant when 0.01 < *p* < 0.05, ** significant when 0.001 < *p* < 0.01, and *** significant when *p* < 0.001.

## 5. Conclusions

Our study identified potential transcription inhibitors that could potentially complement existing cART drugs to address the therapeutic gap in current regimens. Additionally, shifting of the TAR RNA loop towards Cyclin T1 upon molecule binding during molecular simulation studies suggested that targeting the TAR loop and Tat-binding UCU bulge together should be an essential feature of TAR inhibitors to achieve complete viral transcription inhibition.

## Data Availability

Data are contained within the article or Supplementary Material.

## Supplementary Materials

The following supporting information can be downloaded at: https://www.mdpi.com/article/10.3390/ph17010033/s1, Figure S1: Chemical Structure of Molecules used in the study; Figure S2: Normalized densitometry analysis of lead TAR-binding molecules effects on HIV-1 infected T-cells (J1.1); Figure S3: Normalized densitometry analysis of lead TAR-binding molecules effects on HIV-1 infected HIV-1 Infected monocytes derived macrophages (U1 MDMs); Figure S4: Densitometry analysis of Proteins from Biotinylated RNA pulldown Assay; Figure S5: Dose dependent Effect of 110FA on Tat-TAR RNA Interaction in a Biotinylated RNA Pull down assay; Figure S6: Western blot analysis of the lead TAR-binding molecules effects on HEK293T cells; Figure S7: Effect of different cut-off values on contact pair between TAR RNA and Tat in native and 110FA bound state; Figure S8: Molecular Dynamics (MD) Derived Model; Table S1: Residue Contact pairs in native and 110FA bound state; Table S2: Binding Energy output by HDOCK.
