# Supplementary material for: HIV-1 Transcription Inhibition Using Small RNA-Binding Molecules
Source: Pharmaceuticals (Basel). 2023 Dec 25;17(1):33. doi: 10.3390/ph17010033 (PMC10819208; doi:10.3390/ph17010033)
Supplement: Supplementary file 1 [file pharmaceuticals-17-00033-s001.zip › pharmaceuticals-2629250-supplementary.pdf]

Supplementary Information

HIV-1 Transcription Inhibition Using Small RNA binding molecules

Pooja Khatkar [1] , Gifty Mensah [1], Shangbo Ning [2], Maria Cowen [1], Yuriy Kim [1], Anastasia Williams [1], Fardokht A. Abulwerdi [3], Yunjie Zhao [2], Chen Zeng [4], Stuart F. J. Le Grice [3] and Fatah Kashanchi [1,*]

[1] Laboratory of Molecular Virology, School of Systems Biology, George Mason University, Manassas, VA 20110, USA
[2] Institute of Biophysics and Department of Physics, Central China Normal University, Wuhan 430079, China
[3] Basic Research Laboratory, National Cancer Institute, Frederick, MD 21702, USA
[4] Physics Department, The George Washington University, Washington, DC 20052, USA

* Correspondence: fkashanc@gmu.edu; Tel.: +1-703-993-9160; Fax: +1-703-993-7022

Supplementary Information

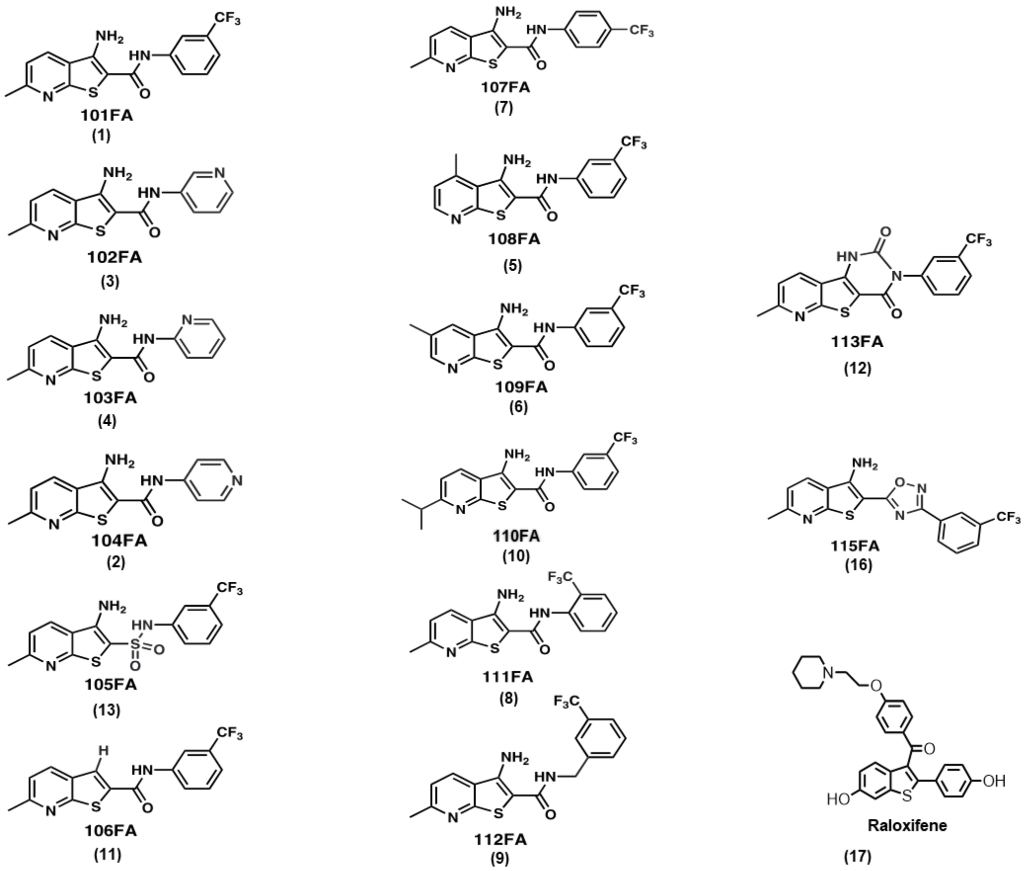

Figure S1. Chemical Structure of Molecules used in the study.

Supplementary Information

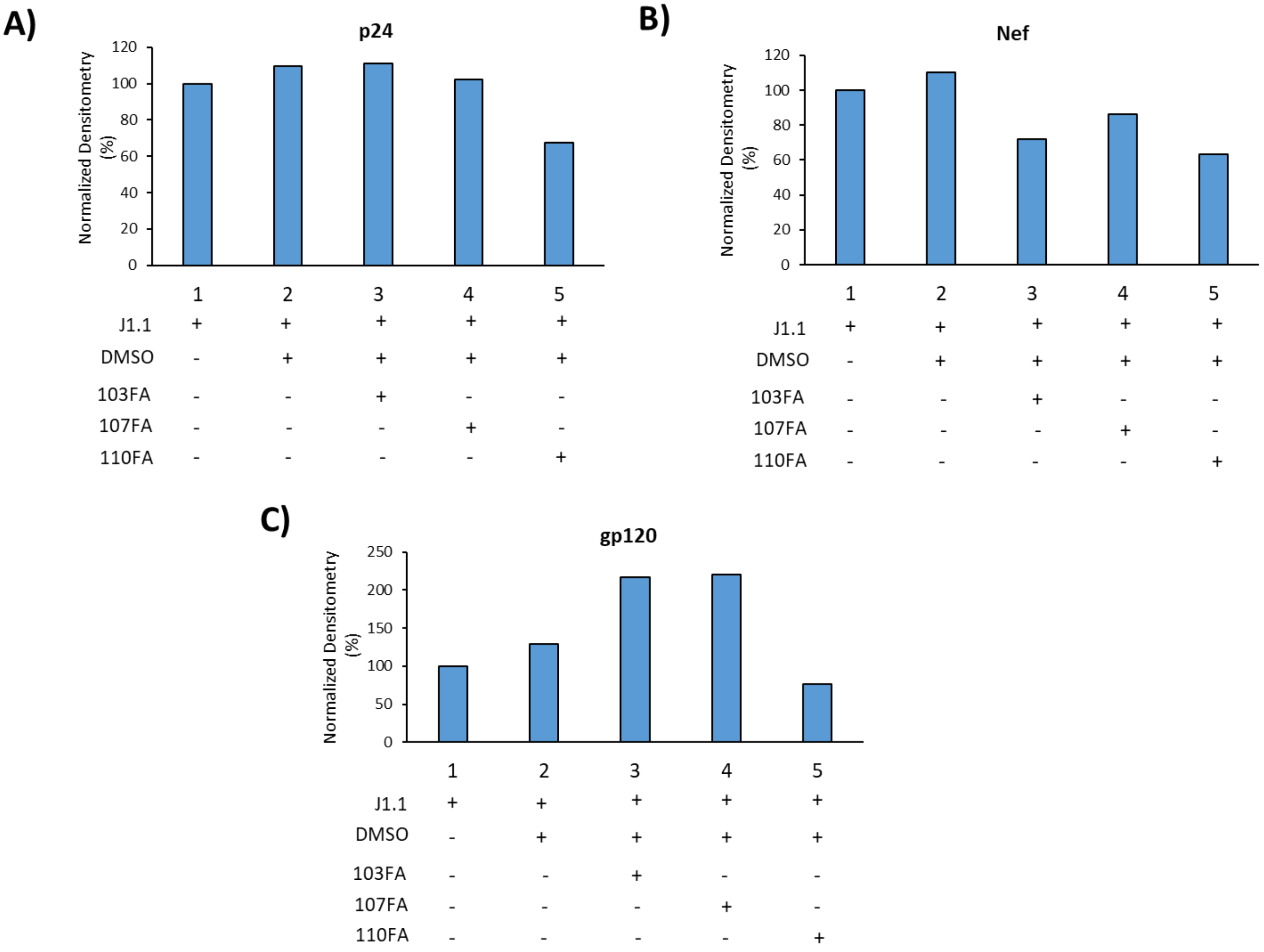

Figure S2. Normalized densitometry analysis of lead TAR-binding molecules effects on HIV-1 infected T-cells (J1.1). (**A**) p24, (**B**) Nef and **(C)** gp120. Densitometry counts

were performed on the Western blot data from Figure 3A, all of which were normalized to Actin levels.

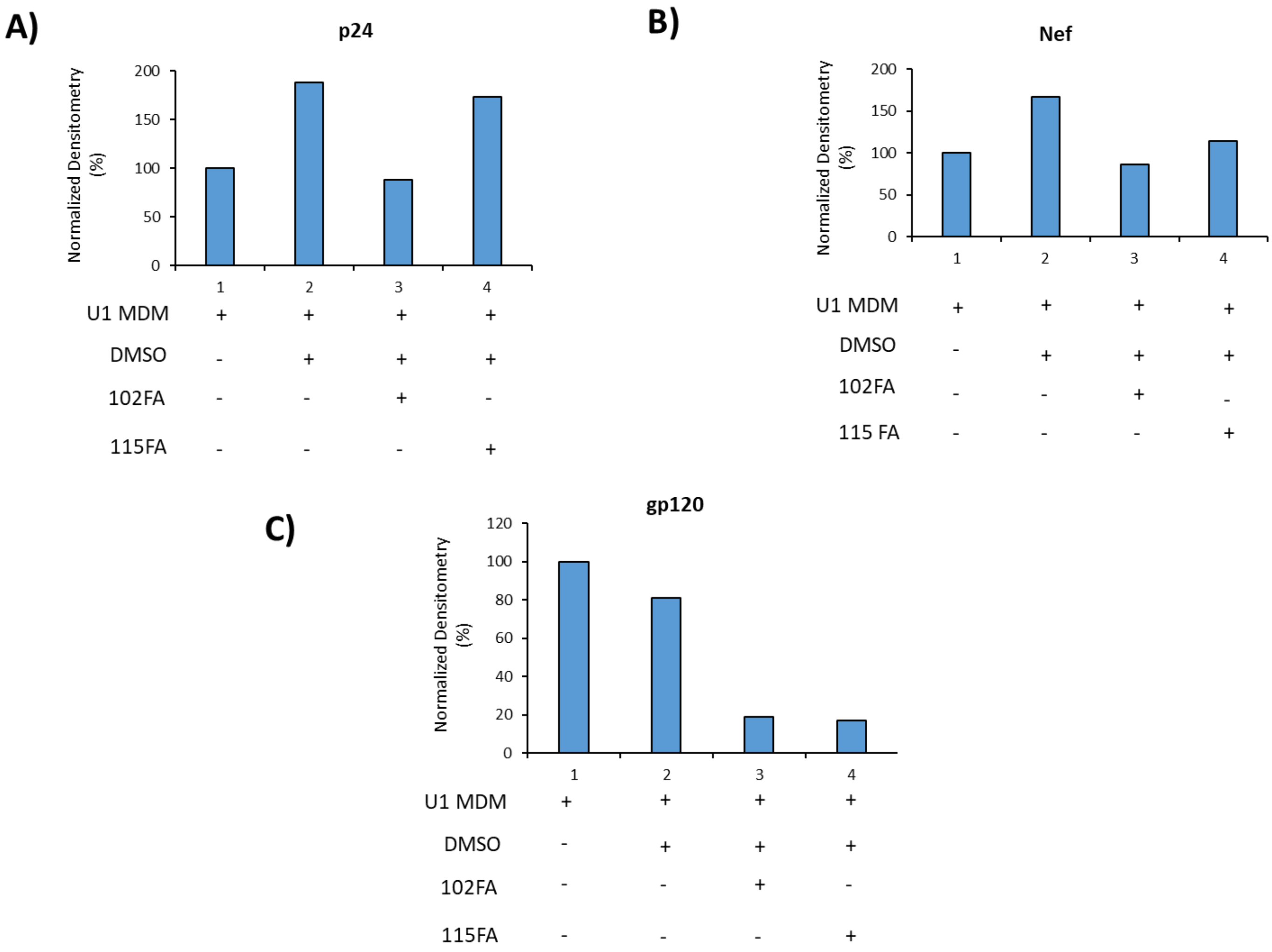

Figure S3. Normalized densitometry analysis of lead TAR-binding molecules effects on HIV-1 infected HIV-1 Infected monocytes derived macrophages (U1 MDMs). (A)

p24, (**B**) Nef, and (**C**)gp120. Densitometry counts were performed on the Western blot data from Figure 3B, all of which were normalized to Actin levels.

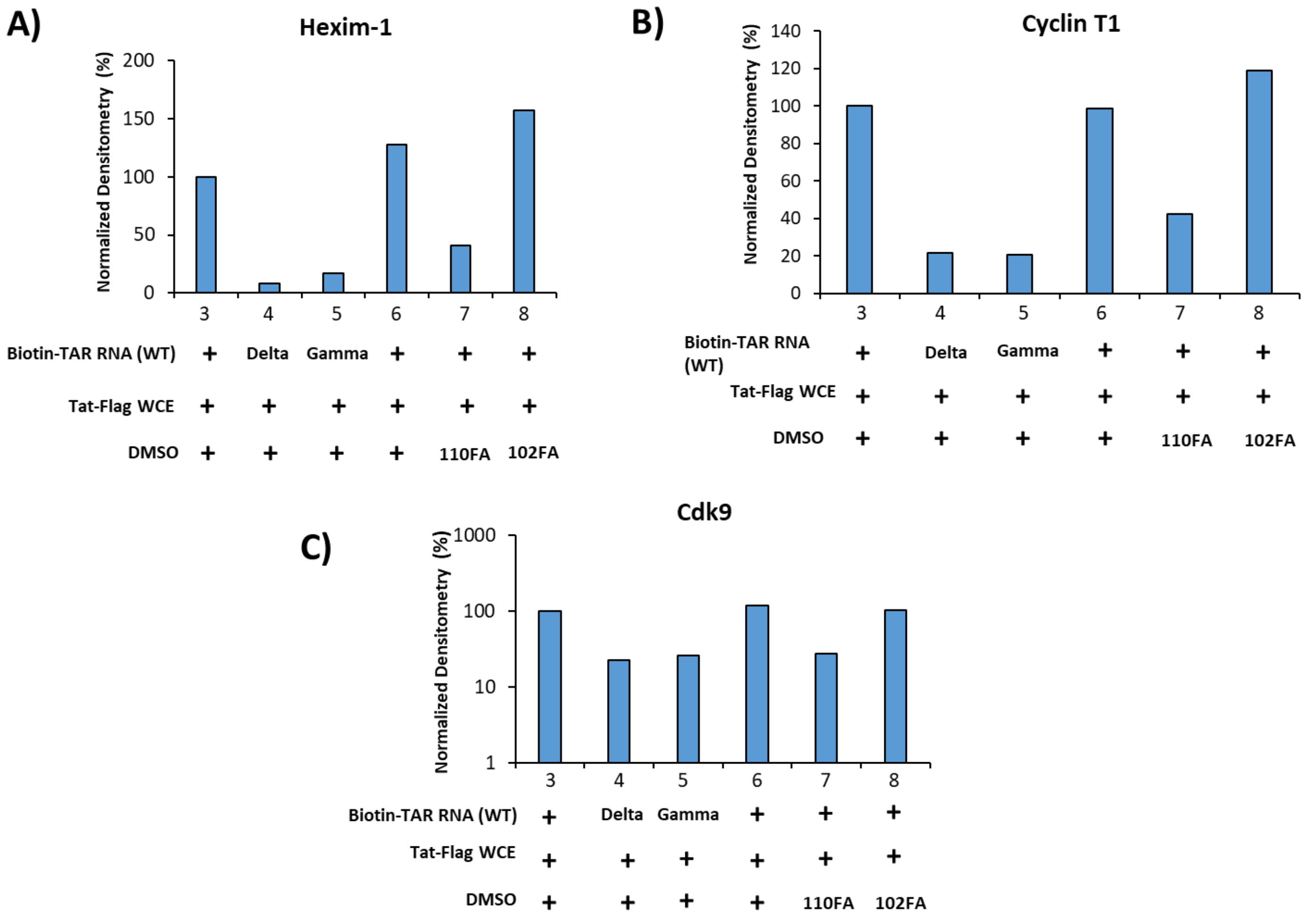

Figure S4. Densitometry analysis of Proteins from Biotinylated RNA pulldown Assay. (**A**) Hexim-1, (**B**) Cyclin T1, (**C**) and Cdk9. Densitometry counts were performed on the Western blot data from Figure 4B.

Supplementary Information

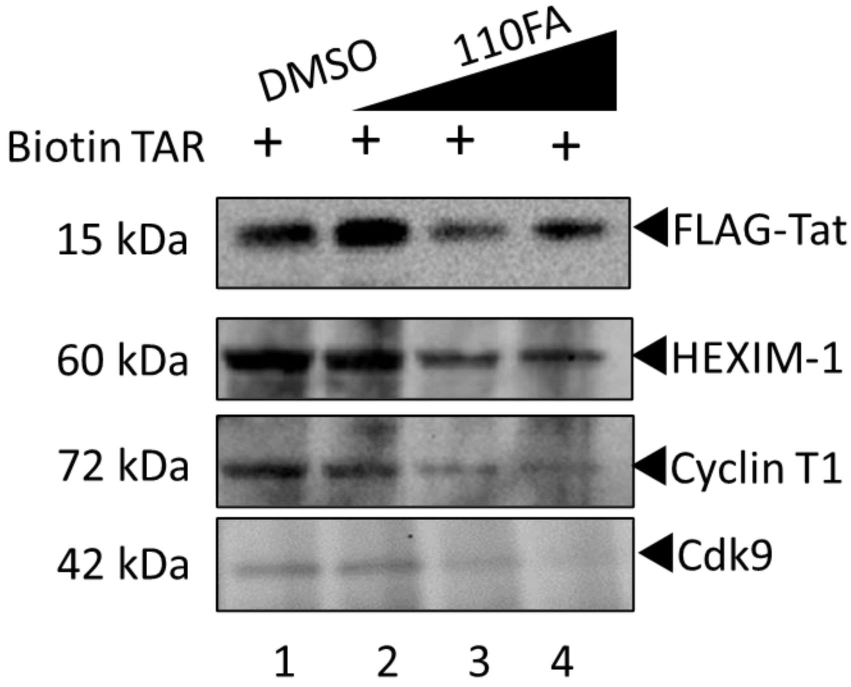

Figure S5. Dose dependent Effect of 110FA on Tat-TAR RNA Interaction in a Biotinylated RNA Pull down assay. Biotinylated RNA pull down assay followed by immunoblot using protein lysate prepared from 293T-cells expressing Flag-Tat and Biotin- WT TAR RNA. After in-cubation, the mixture was treated with 1, 5 and 10μM of the TAR RNA binding molecules 110FA. Coupled proteins were eluted out from beads with Laemmli buffer, run on SDS-PAGE and detected with Anti-Flag, HEXIM-1, Cyclin T1, Cdk9 antibodies.

Supplementary Information

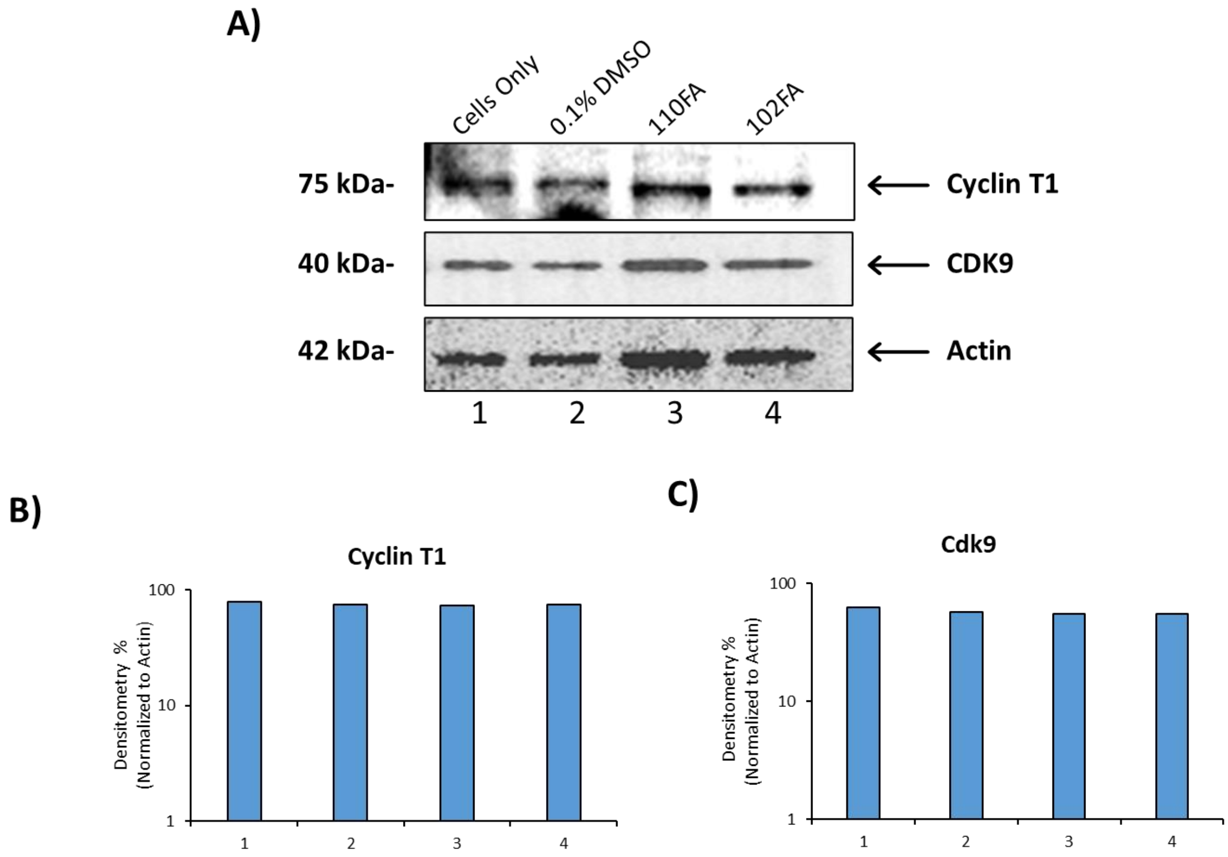

Figure S6. Western blot analysis of the lead TAR-binding molecules effects on HEK293T cells. A) The two lead TAR-binding molecules 110FA and 102FA were used to treat HEK293T cells at 1 μM each at 0 hr and 48hrs timepoints. After 72 h of treatment,

whole cell lysates were generated, run on Western blots, and probed with antibodies against HIV-1 viral proteins Cyclin T1 and Cdk9. Additionally, β-Actin was probed as a control. Densitometry counts were performed on the Western blot data for Cyclin T1 **(B)** and Cdk9 **(C)** all of which were normalized to Actin levels.

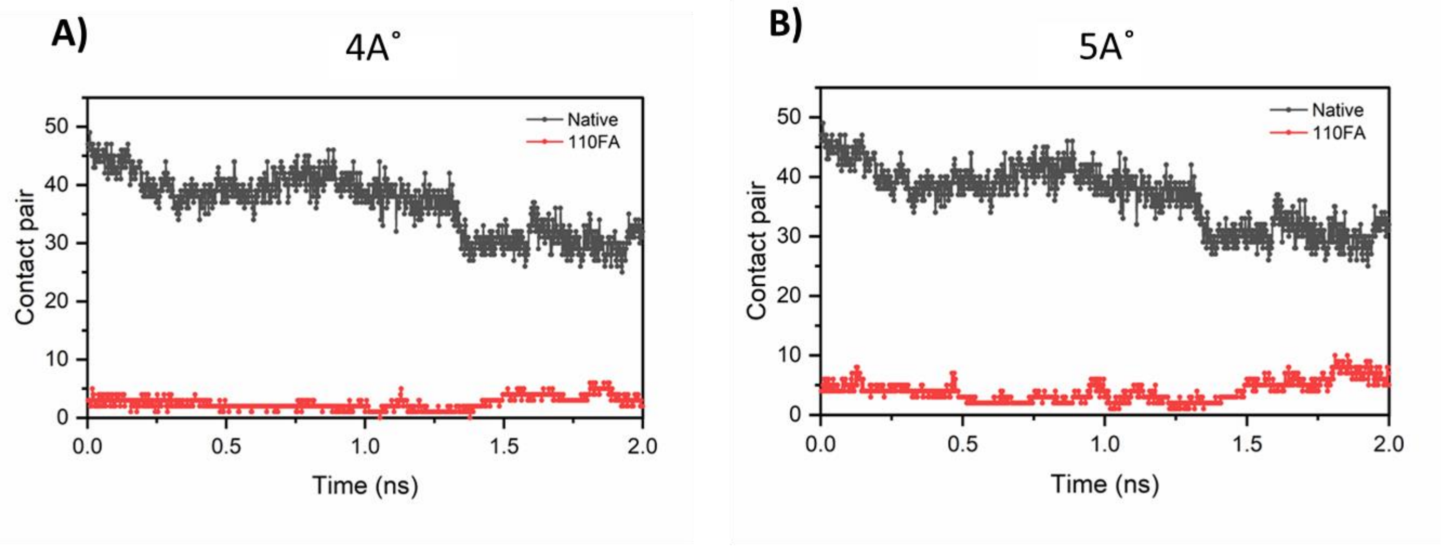

Figure S7. Effect of different cut-off values on contact pair between TAR RNA and Tat in native and 110FA bound state. Contact pair between TAR RNA and Tat protein was calculated in complex with and without 110FA at 4A˚ **(A)** and 5A˚ **(B).**

Supplementary Information

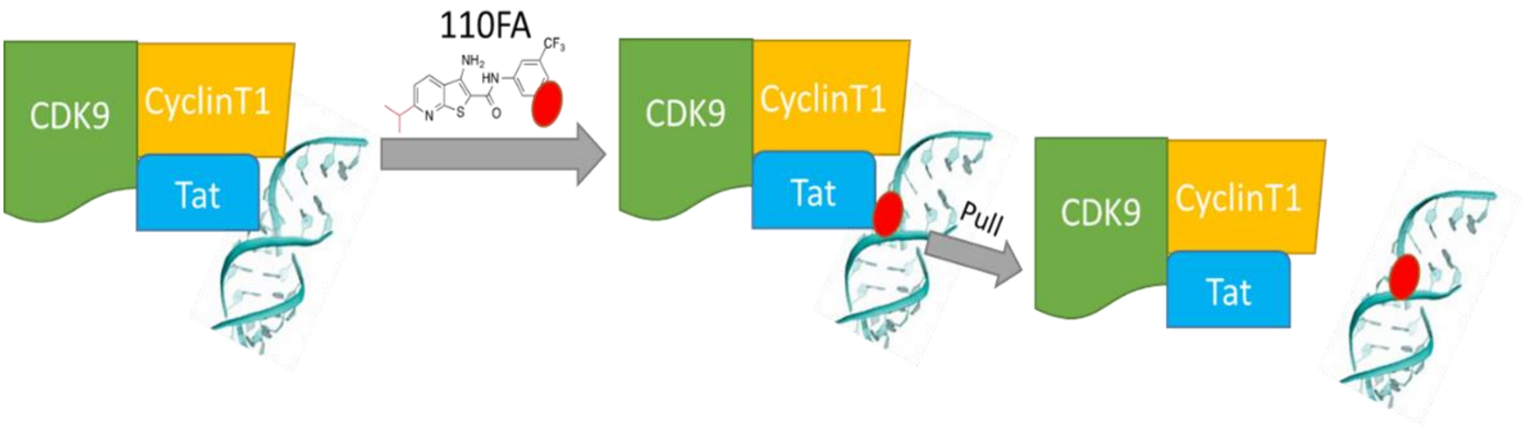

Figure S8. Molecular Dynamics (MD) Derived Model.

Supplementary Information

Supplementary Table S1 : Residue Contact pairs in native and 110FA bound state.

1. **Native Contacts (Figure 9A)**

**Cdk9-Tat (PDB ID: 6CYT):**
S175-K9
R172, A173, F174-W11
S175, A177, N183-K12
**CyclinT1-TAR (PDB ID: 6CYT):**
R251-U31
W258-G32
R259-U31, G32

2. **List of "decreased" contacts with 110FA (Figure 9B) are:**

**Tat-TAR RNA:**
G19-K50, K51, R52
A20-K50, R56
G21-K50, R56
C22-R52, R53, R56, R57
U23-R52, R53, Q54
C24-R54
C26, U27- R54

Supplementary Information

Supplementary Table S2. Binding Energy output by HDOCK.

	<u>Without Complex</u>		<u>With Complex</u>		
**Molecules**	102FA	110FA	102FA	110FA	**Binding energy (Kcal/mol)**
**1**	-99.51	-104.14	-153.32	-146.06	
**2**	-95.34	-103.18	-149.37	-138.46	
**3**	-89.81	-101.12	-140.73	-133.16	
**4**	-86.6	-99.38	-137.75	-129.97	
**5**	-85.95	-96.73	-137.46	-128.87	
**6**	-82.85	-89.56	-134.96	-128.13	
**7**	-70.39	-85.04	-131.61	-126.76	
**8**	-69.92	-84.91	-128	-125.48	
**9**	-68.86	-83.43	-122.07	-124.32	
**10**	-68.13	-80.33	-120.02	-123.55	
